# Role of long- and short-range hydrophobic, hydrophilic and charged residues contact network in protein’s structural organization

**DOI:** 10.1186/1471-2105-13-142

**Published:** 2012-06-21

**Authors:** Dhriti Sengupta, Sudip Kundu

**Affiliations:** 1 Department of Biophysics, Molecular Biology & Bioinformatics, University of Calcutta, 92 APC Road, Kolkata-700009, India

**Keywords:** Protein contact network, Largest cluster transition, Assortativity, Clustering coefficient, Cliques

## Abstract

**Background:**

The three-dimensional structure of a protein can be described as a graph where nodes represent residues and the strength of non-covalent interactions between them are edges. These protein contact networks can be separated into long and short-range interactions networks depending on the positions of amino acids in primary structure. Long-range interactions play a distinct role in determining the tertiary structure of a protein while short-range interactions could largely contribute to the secondary structure formations. In addition, physico chemical properties and the linear arrangement of amino acids of the primary structure of a protein determines its three dimensional structure. Here, we present an extensive analysis of protein contact subnetworks based on the London van der Waals interactions of amino acids at different length scales. We further subdivided those networks in hydrophobic, hydrophilic and charged residues networks and have tried to correlate their influence in the overall topology and organization of a protein.

**Results:**

The largest connected component (LCC) of long (LRN)-, short (SRN)- and all-range (ARN) networks within proteins exhibit a transition behaviour when plotted against different interaction strengths of edges among amino acid nodes. While short-range networks having chain like structures exhibit highly cooperative transition; long- and all-range networks, which are more similar to each other, have non-chain like structures and show less cooperativity. Further, the hydrophobic residues subnetworks in long- and all-range networks have similar transition behaviours with all residues all-range networks, but the hydrophilic and charged residues networks don’t. While the nature of transitions of LCC’s sizes is same in SRNs for thermophiles and mesophiles, there exists a clear difference in LRNs. The presence of larger size of interconnected long-range interactions in thermophiles than mesophiles, even at higher interaction strength between amino acids, give extra stability to the tertiary structure of the thermophiles. All the subnetworks at different length scales (ARNs, LRNs and SRNs) show assortativity mixing property of their participating amino acids. While there exists a significant higher percentage of hydrophobic subclusters over others in ARNs and LRNs; we do not find the assortative mixing behaviour of any the subclusters in SRNs. The clustering coefficient of hydrophobic subclusters in long-range network is the highest among types of subnetworks. There exist highly cliquish hydrophobic nodes followed by charged nodes in LRNs and ARNs; on the other hand, we observe the highest dominance of charged residues cliques in short-range networks. Studies on the perimeter of the cliques also show higher occurrences of hydrophobic and charged residues’ cliques.

**Conclusions:**

The simple framework of protein contact networks and their subnetworks based on London van der Waals force is able to capture several known properties of protein structure as well as can unravel several new features. The thermophiles do not only have the higher number of long-range interactions; they also have larger cluster of connected residues at higher interaction strengths among amino acids, than their mesophilic counterparts. It can reestablish the significant role of long-range hydrophobic clusters in protein folding and stabilization; at the same time, it shed light on the higher communication ability of hydrophobic subnetworks over the others. The results give an indication of the controlling role of hydrophobic subclusters in determining protein’s folding rate. The occurrences of higher perimeters of hydrophobic and charged cliques imply the role of charged residues as well as hydrophobic residues in stabilizing the distant part of primary structure of a protein through London van der Waals interaction.

## Background

Proteins are important biomolecules having a large number of structural and functional diversities [1]. It is believed that these 3D structural, and hence functional, diversities of proteins are imprinted in the primary structure of proteins. While the primary structure of a protein is a linear arrangement of different amino acids connected with their nearest neighbours through peptide bonds in 1D space, the 3D structure can be considered as a complex system emerged through the interactions of its constituent amino acids. The interactions among the amino acids within a protein can be presented as an amino acid network (often called as protein contact network) in which amino acids represent the nodes and the interactions (mainly non-bonded, non-covalent) among them represent the undirected edges. This representation provides a powerful framework to uncover the general organized principle of protein contact network and also to understand the sequence structure function relationship of this complex biomolecule [2-5]. Analysis of different topological parameters of protein contact networks help researchers to understand the various important aspects of a protein including its structural flexibility, key residues stabilizing its 3D structure, folding nucleus, important functional residues, mixing behavior of the amino acids, hierarchy of the structure, etc [6-12]. A web-server AminoNet has recently been launched to construct, visualize and calculate the topological parameters of amino acid network within a protein [13].

Researchers have also studied the role of inter-residue interactions at different length scales of primary structure in protein folding and stability [14-20]. Long-range interactions are said to play a distinct role in determining the tertiary structure of a protein, as opposed to short-range interactions, which could largely contribute to the secondary structure formations [14,15]. Bagler and Sinha have concluded that assortative mixing (where, the nodes with high degree have tendency to be connected with other high degree nodes) of long-range networks may assist in speeding up of the folding process [21]. They have also observed that the average clustering coefficients of long-range scales show a good negative correlation with the rate of folding of proteins. It should be clearly noted that while the long and short-range interactions are determined by the positions of amino acids in primary structure, the contact networks are determined by the positions of amino acids’ in 3D space.

When a protein folds in its native conformation, its native 3D structure is determined by the physico-chemical nature of its constituent amino acids. The dominance of hydrophobic residues in protein folding is already shown in [22-24]. The role of long-range hydrophobic clusters in folding of (*α*/*β*)_8_ barrel proteins [17] and in the folding transition state of two-state proteins is also reported in [19]. Poupon and Mornon have shown a striking correspondence between the conserved hydrophobic positions of a protein and the intermediates formed during its initial stages of folding constituting the folding nucleus [25]. We too have performed a comparative topological study of the hydrophobic, hydrophilic and charged residues contact networks and have shown that hydrophobic residues are mostly responsible for the overall topological features of a protein [12]. Very recently, we have studied how the topological parameters of amino acids within a protein contact network depend on the their physico chemical properties [26].

However, the topology of protein contact subnetworks based on physico chemical properties of amino acids and at the same time, at different length scale has not been studied extensively. In our present study, we have constructed and analyzed protein contact networks at two different length scales, long-range and short- range, for a large number of proteins covering all classes and folds. These long and short-range amino acids contact networks have been further divided into subnetworks of hydrophobic, hydrophilic and charged residues.

Here, we have studied the transition of largest cluster sizes; the mixing behaviour of nodes; overall cliquishness as well as preference of specific types of cliques (subgraph where every pair of vertices are connected by an edge) over others in different subnetworks. We observe that the transition behaviours of long-range networks and short-range networks are different and the former have higher similarity with all-range networks. Comparison of the homologs of mesophilic and thermophilic proteins show that there exist a difference in their long-range networks. While the mixing behaviour of amino acids within all-range contact network is reflected in their long- and short-range subnetworks, the hydrophobic subnetworks have a major significant contribution in determining the overall mixing property of long-range networks. We also demonstrate the higher occurrence of hydrophobic residues’ cliques in all- and long-range networks. On the other hand, cliques of charged residues are over-represented in short-range networks. There also exist higher perimeter of charged residues cliques with three vertices (in addition to hydrophobic cliques), which in turn, indicate to the importance of charged residues in bringing and stabilizing the distant part of primary structure in 3D space.

## Methods

### Construction of amino acid networks

Primary structure of a protein is a linear arrangement of twenty different types of amino acids in one-dimensional space where any amino acid is connected with its nearest neighbours through peptide bonds. But when a protein folds in its native conformation, distant amino acids in the one-dimensional chain may also come close to each other in 3D space, and hence, different non-covalent interactions are possible among them depending on their orientations in 3D space. Considering the amino acids as nodes and the London van der Waals’ interactions (which satisfy the condition given below) among them as edges, we construct protein contact network (PCN).

#### Interaction strength between amino acids

Strength of interaction between two amino acid side chains is evaluated as a percentage given in [4] by:

(1) $$I_{\mathrm{ij}}=\frac{n_{\mathrm{ij}}}{\sqrt{N_{i}\times N_{j}}}\times 100$$

where, *n*_*ij*_ is the number of distinct interacting pairs of side-chain atoms between the residues *i* and *j*, which come within a distance of 5A^∘^(the higher cutoff for attractive London–van der Waals forces [27]) in the 3D space. *N*_*i*_ and *N*_*j*_ are the normalization factors for the residues *i* and *j*, respectively. We have determined the normalization factors *N*_*i*_ for all 20 residue types using the method described in [3] and given below.

(2) $$N_{i}=\overset{p}{\underset{j=1}{\sum }}\frac{\mathrm{MAXM}(\mathrm{TYPE}(i_{k}))}{p}$$

The number of interaction pairs including main-chain and side-chain made by residue type *i* with all its surrounding residues in a protein *k* is evaluated. *MAXM*(*TYPE*(*i*_*k*_)) is considered by the maximum number of interactions make by residue *i* in protein *k*. In our case, *k* varies from 1 to 495 (the size of our data set). The normalization factors take into account the differences in the sizes of the side chains of the different residue types and their propensity to make the maximum number of contacts with other amino acid residues in protein structures [3].

#### Existence of edge between amino acid nodes

An important feature of such a graph is the definition of edges based on the normalized strength of interaction between the amino acid residues in proteins. Once *I*_*ij*_ is evaluated for all pairs of amino acid residues, a cutoff value (*I*_*min*_) is chosen. Any pair of amino acid residues (*i* and *j*) with an interaction strength of *I*_*ij*_, are connected by an edge if *I*_*ij*_>*I*_*min*_. This cutoff (*I*_*min*_) is varied from 0%(> 0% is referred as 0%) to 10%. Thereafter, PCNs are constructed for all the proteins present in our data set at these varying cutoffs. As the interaction cutoff increases from 0% to 10%, the number of edges in the PCNs decreases; because, at higher cutoff, the number of nodes making the higher number of interactions is less. Very few numbers of amino acids sustain interactions at 10% cutoff. It should be mentioned that the definition of amino acid interaction is purely based on the number of distance-based London van der Waals’ contacts between two amino acid residues.

#### PDB structures used

A total of 3,087 non-redundant proteins were retrieved from the protein data bank [28] that fulfill the following criteria: 1) Maximum percentage identity: 30, 2) Resolution: ≤ 3.0, 3) Maximum R-value: 0.3, 4) Sequence length: 300-10,000, 5) CA only entries: excluded, 6) Non X-ray entries: excluded and 7) CULLPDB by chain. We should mention that proteins with less than 300 amino acids are avoided in this study to get subclusters (from different subnetworks) of reasonable size. Subclusters with less than 30 amino acids are not enough for study of topological parameters.

A set of 3,087 proteins meet up the above mentioned criteria. From this set, we removed all those proteins for which the atomic coordinates of any amino acid are missing. The protein contact networks that we generate are totally based on atomic distances of the amino acids, so missing amino acids or atomic coordinates may give erroneous values of different network parameters (degree, clustering coefficient, etc). Finally, we obtained a set of 495 proteins (PDB codes listed in Additional file 1) for our analysis.

#### Long-range, short-range and all-range protein contact subnetworks

We have constructed the long-range interaction network (LRN), short-range interaction network (SRN) and all-range interaction network (ARN). If any amino acid *i* has an interaction with any other amino acid *j*, whether this would be a part of the LRN or SRN depends on the distance *x*=|*i*−*j*| between the *i*^*th*^ and *j*^*th*^ amino acids in the primary structure. If *x*>10, LRN is produced, while if *x*≤10, a SRN is produced [5,12,26]. It is clear that *x*>0 will provide ARN.

#### Hydrophobic, hydrophilic and charged residues subnetworks

It is also known that each of the 20 amino acids within a protein has different side chain and different physico-chemical properties. Based on it, the 20 amino acid residues are grouped into three major classes: hydrophobic (F, M, W, I, V, L, P, A), hydrophilic (N, C, Q, G, S, T, Y), and charged (R, D, E, H, K)[12]. We have generated hydrophobic networks (BN) where the hydrophobic residues are considered as nodes and link between them is established if their interaction strength exceeds a particular threshold (as defined earlier). Hydrophilic networks (IN), charged networks (CN) and all amino acid networks (AN) are constructed similarly. We should once again mention that the BNs, INs and CNs generated here are based only on the Van der Walls forces. The networks thus formed have more than one subnetwork, with the number of nodes varying over a wider range.

### Network parameters

Each of the networks is represented as an adjacency matrix. Any element of the adjacency matrix (A), connecting the *i*^*th*^ and *j*^*th*^ nodes, is given as: *a*_*ij*_ = 1, if *i*≠*j* and nodes *i* and *j* are connected by an edge, the value is 0 if *i*≠*j* and nodes *i* and *j* are not connected or if *i*=*j*.

#### Mixing behaviour of nodes

To study the tendency for nodes in networks to be connected to other nodes that are like (or unlike) them, we have calculated the Pearson correlation coefficient (*r*) of the degrees at either ends of an edge. Its value has been calculated using the expression suggested by Newman [29] and is given as

(3) $$r=\frac{M^{-1}\underset{i}{\sum }j_{i}k_{i}-{\left[M^{-1}\underset{i}{\sum }0.5(j_{i}+k_{i})\right]}^{2}}{M^{-1}\underset{i}{\sum }0.5(j_{i}^{2}+k_{i}^{2})-{\left[M^{-1}\underset{i}{\sum }0.5(j_{i}+k_{i})\right]}^{2}}$$

Here *j*_*i*_ and *k*_*i*_ are the degrees of the vertices at the ends of the *i*^*th*^edge, with *i*=1,*.....M*. The networks having positive and negative *r* values are assortative and disassortative, respectively. In addition, the value of this parameter (*r*) gives a quantitative estimation of the mixing behaviour of nodes in a network.

#### Clustering coefficients

The clustering coefficient (*C*) is a measure of local cohesiveness. (*C*_*i*_) of a node *i* is the ratio between the total number of links actually connecting its nearest neighbors and the total number of possible links between the nearest neighbors of node *i*. In other words,(*C*_*i*_) enumerates the number of loops of length three maintained by a node *i* and its interconnected neighbors. It is given by

(4) $$C_{i}=\frac{2e_{i}}{k_{i}(k_{i}-1)}$$

Here *e*_*i*_ is the total number of edges actually connecting the *i*^*th*^node’s nearest neighbors and *k*_*i*_is the number of neighboring nodes of node *i*.

#### Largest Connected Component

After the adjacency matrices are constructed at different cutoffs of varying strengths of interaction, they are subsequently subjected to depth first search method [30] to identify their distinct clusters and cluster forming nodes. The giant cluster (defined here as “Largest Connected Component” or LCC) is the largest group of connected nodes in a network that are reachable to each other directly or indirectly. The size of the LCC in a network (in terms of the number of amino acid residues) depends on the connection (edges) among amino acid nodes and the existence of edge depends on the interaction strength cut-off. Thus, the size of LCC becomes a function of *I*_*min*_cut-off.

We have determined the largest connected components and their sizes from adjacency matrices formed at varying cutoffs of strengths of interaction. The sizes of largest cluster are normalized with respect to the total number of residues in the protein, so that it is no more dependent on the size of the protein.

## Results and discussion

We have constructed and analyzed hydrophobic (BN), hydrophilic (IN), charged (CN) and all (AN) residues’ London van der Waals contact networks at three different length scales [long-range interaction networks (LRNs), short-range interaction networks (SRNs) and all-range interaction networks (ARNs)] for each of the 495 proteins at different interaction strength (*I*_*min*_) cutoffs (see Methods).

Earlier studies showed that the Largest Connected Component (LCC) is a very important parameter in network analysis, it provides information on the nature and connectivity of the network [4,31]. The normalized size of LCC when plotted as a function of *I*_*min*_value, undergoes a transition for all proteins , irrespective of their sizes or folds. The *I*_*min*_ value at which the size of LCC is half of the size at *I*_*min*_=0% is termed as *I*_*critical*_[4,31]. It is also reported that the values of *I*_*critical*_fall within a narrow range for proteins of all sizes and folds [4].

**Figure 1:**
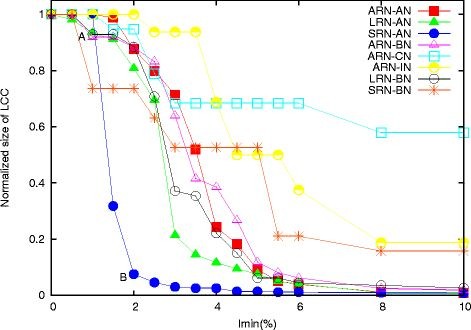
**Transition profile of different subnetworks.** The normalized size of largest connected component (LCC) is plotted as a function of *I*_*min*_for different subnetworks in a representative protein (PDB code: 1A0C). The subnetworks are - All-range all-residue network (ARN-AN), Long-range all-residue network (LRN-AN), Short-range all-residue network (SRN-AN), All-range hydrophobic-residue network (ARN-BN), All-range hydrophilic-residue network (ARN-IN), All-range charged-residue network (ARN-CN), Long-range hydrophobic-residue network (LRN-BN) and Short-range hydrophobic-residue network (SRN-BN).

Here, we have first studied the nature of transition of different subclusters (LRN, SRN, ARN and BN, IN, CN, AN). We have plotted the normalized size of the LCC as a function of *I*_*min*_ (from *I*_*min*_ = 0% to 10%) for different subnetworks (Figure 1 and Additional file 2).

### Transitions of largest clusters’ sizes depend on length scale of the networks

Results indicate that the nature of transition in ARN-AN is closer to LRN-AN than SRN-AN (Figure 1). As expected, in ARN-ANs, the largest cluster include all of the residues in the protein at *I*_*min*_= 0%. The transitions take place within a narrow range (2% - 5.5%), with *I*_*critical*_ varying from nearly 3% to 4.5% in approximately 90% proteins (Figure 1). However, in LRN-ANs, the transition begins from a slight lower cutoff, and the process of transition in LCC is faster than ARN-ANs but slower than SRN-ANs. In LRN-ANs, the *I*_*critical*_values vary from 1.5% to 3% in approximately 88% proteins. On the other hand, the transitions in SRN-ANs are very steep; and in approximately 86% proteins, the values of *I*_*critical*_vary from 1% to 1.5%. In SRNs, the clusters are highly connected at lower *I*_*min*_cutoffs, infact, the average cluster size of SRN-ANs (almost same as ARN-ANs) is higher than LRN-ANs at *I*_*min*_ = 0% (Table 1).

**Table 1:** **Average cluster size, average Pearson correlation coefficient (〈** ***r*** **〉) and average clustering coefficients (〈** ***C*** **〉) of hydrophobic (BN), hydrophilic (IN), charged (CN), and all-amino-acids (AN) networks at different length scales viz. the long-range (LRN), short-range (SRN) and all-range (ARN) interaction networks are listed forI**_***min***_= 0

**Length scale**	**Type**	**Avg cluster size**	**〈** ***r*** **〉**	**〈** ***C*** **〉**
LRN	BN	101.59 ± 53.66	0.13 ± 0.10	0.24 ± 0.05
	IN	44.16 ± 13.03	-0.04 ± 0.19	0.14 ± 0.06
	AN	350.5 ± 134.77	0.17 ± 0.07	0.16 ± 0.03
SRN	BN	38.55 ± 11.10	-0.11 ± 0.17	0.29 ± 0.08
	AN	430.93 ± 145.06	0.21 ± 0.06	0.35 ± 0.03
ARN	BN	156.59 ± 70.75	0.27 ± 0.08	0.39 ± 0.03
	IN	68.38 ± 41.33	0.15 ± 0.15	0.29 ± 0.06
	CN	47.42 ± 18.34	0.14 ± 0.16	0.27 ± 0.07
	AN	436.28 ± 141.01	0.30 ± 0.04	0.35 ± 0.01

Thus, the above results clearly indicate -(i) sharp transition of SRNs in comparison to LRN and ARNs, (ii) early transition of SRNs, and (iii) more similar transition of LRNs and ARNs. The steep transition in SRN-ANs is attributed to the fact that it has a chain like structure at *I*_*min*_=0% (Additional File 3), and as *I*_*min*_increases, the loss of a specific contact in this chain-like cluster has a high probability to break the chain, thus quickly generating a larger number of clusters. On the other hand, the early onset of transition in SRN-ANs (Figure 1) is attributed to the fact that they have a significant lower strength of interaction (*I*_*ij*_) than LRN-ANs (2.56 and 2.86, respectively, with p < 0.05). However, we should mention that the average degree of SRN-ANs is higher than LRN-ANs at *I*_*min*_=0% (4.03 and 3.93, respectively).

On the other hand, the LRN and ARN at *I*_*min*_=0% do not have chain like structures (Additional File 3) and thus they are more resistant to the elimination of edges as *I*_*min*_ increases. This is also one of the reasons why the transitions of LRN and ARN are more similar. Furthermore, in ARN-ANs, at lower *I*_*min*_cutoff, when all of the residues are connected in a single large cluster, both the long- and short-range interactions are involved in it. But as we increase the cutoff, the contribution from short-range interactions decreases more rapidly than long-range interactions. And thereafter (at higher *I*_*min*_ cut off), the residues in the protein network are mainly connected by the long-range interactions. So, these explain the similar transition nature of LCC in ARN-ANs and LRN-ANs.

It is also well established that the long-range interactions (interactions among amino acids distantly placed in primary structure) stabilizes the tertiary structural integration of a protein. Thus, the similar transition behaviour of LRN and ARN is also expected. The similarity in transition profile of long-range and all-range network’s LCC in proteins suggest that long-range interactions are guiding the overall topology and stability of the tertiary structure of a protein. At the same time, we want to give emphasis on another point described below. The interaction strength gives a clear measure of how the amino acids are connected and tightly bound within a protein, which in turn is related to the packing and stability of a protein. The tertiary structure is mainly stabilized through interactions among amino acids placed at long distant in the primary structure. Thus, the existence of comparative larger size LCC in LRNs at higher *I*_*min*_ suggests that a protein may need larger amount of possible non-covalent interactions (in addition to others) in bringing and holding together distant part of the primary structure of a protein in 3D space.

The difference in transition profiles of LRN and SRN clearly also indicate that the cooperativities of their transitions are different. One may be interested to compare the cooperativity indexes of those transitions. The shape of the LCC size versus *I*_*min*_ curve can be expressed in the terms of the ratio of the *I*_*min*_ cutoff at which the transitions begins and the *I*_*min*_cutoff at which the clusters just break down into many small sub-clusters (for example, points A and B as marked in SRN-AN of Figure 1). This ratio is called the cooperativity index (CI) [32]. Higher CI value suggests more cooperativity. Without any numerical calculation, just from the nature of transition profiles, it is very much clear that the CI values for SRN-ANs are comparatively very high than those of LRN-ANs and ARN-ANs. When we calculate it in a representative protein 1A0C, SRN-AN show the highest average CI value (0.53), which is approximately 1.5 times of CI values of LRNs (0.35) and ARNs (0.31). We want to mention that a more rigorous general method is needed to define the point A and B of Figure 1.

#### Transition of hydrophobic subcluster is similar to that of all amino acids network

We have also studied how the sizes of the largest clusters vary in the ARN-BNs, ARN-INs and ARN-CNs. Here, we find that ARN-BNs have a transition nature more inclined towards the ARN-ANs (Figure 1). The transition takes place in exactly the same range of ARN-ANs; *I*_*critical*_ varies from 2.5% to 4.5%. On the contrary, ARN-INs and ARN-CNs don’t show any single state transition throughout (Figure 1). Interestingly, when comparing LRN-BNs and SRN-BNs, the nature of transition in LRN-BNs are more closer to ARN-ANs (*I*_*critical*_∼ 3) than SRN-BNs which do not show a clear phenomenon of single state transition (Figure 1).

The above results clearly indicate the predominant role of hydrophobic subclusters in shaping the transition behaviour of long-range and all range all amino acids network.

#### Thermophilic and mesophilic show differences in their long-range transition

We have also studied the variation of LCC in 12 pairs of mesophilic and their corresponding thermophilic proteins (PDB IDs are taken from [4]). Comparing the size of LCC of mesophilic and thermophilic proteins at different *I*_*min*_, Brinda *et al* have observed the larger size of LCC in thermophilics and this gives possible explanation for their higher stability [4].

**Figure 2:**
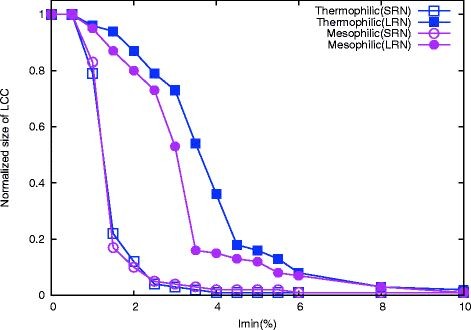
**Difference in transition profiles of thermophilic and mesophilic proteins at different length scales.** The normalized size of largest connected component (LCC) is plotted as a function of *I*_*min*_in thermophilic (PDB code: 1XYZ) and mesophilic (PDB code: 2EXO) protein at long-range and short-range network.

Here, we have studied the transition of LCC for SRNs, LRNs and ARNs separately (Figure 2). While the nature of transitions of LCC’s sizes are same in SRNs for thermophiles and mesophiles, there exist a clear difference in LRNs. The *I*_*critical*_values for SRNs lies between1-1.5 in both thermophiles and mesophiles. But, in LRNs, the values of *I*_*critical*_(lies between 3.5-4) for thermophiles are higher than those of mesophiles (*I*_*critical*_ lies between 3-3.5). The presence of larger size of interconnected long-range interactions in thermophiles than mesophiles, even at higher *I*_*min*_cut-off, give extra stability to the tertiary structure of the thermophiles.

Brinda *et al*[4] showed that at higher *I*_*min*_the size of LCC of ARN in thermophilic is higher than that of mesophilic and thus providing extra stability to the thermophilic protein. They have not studied the transition of long and short -range networks separately. However, Gromiha [33] clearly predicted that the residues occurring in the range of 31-34 residues apart in the sequence contribute significant long-range contacts to the stability of thermophilic proteins. They also showed that the thermophiles have more residue pairs than mesophilics. Here, our results confirm the previous observations; in addition, it shows that the thermophiles do not have only the higher number of residue pairs in long-range interactions, they have also larger cluster of connected residues at higher *I*_*min*_ than their mesophilic counterparts. This observation also indicates that there exist higher interaction strengths among the amino acid nodes of these thermophilic long-range clusters.

### Mixing behaviour of the amino acid nodes

Next, we shall discuss the mixing behavior of nodes in different subclusters and try to find whether an amino acid with higher number of connections have tendency to be connected with another amino acid with higher degree or not. This, in turn, will give also an idea whether the probability of connections of any amino acid with other is random or it has any preference. In our earlier work, we showed assortative mixing behaviour of the hydrophobic residues in overall protein structure [12]. Here, we extend those studies in different subnetworks to get an idea of their individual nature and also their relative contribution in fixing the mixing behaviour of amino acids in overall protein.

To understand this mixing behaviour, we have calculated Pearson correlation coefficient (*r*) of the networks (for details see Methods). Depending on the mixing behavior of nodes, a network is either of two types – assortative ( +*r* value) or disassortative ( -*r* value). A network is said to be assortative, if the high-degree nodes in the network tend to be connected with other high-degree nodes and disassortative when the high-degree nodes tend to connect to other low-degree nodes.

#### Different length scales networks (LRN, SRN and ARN) are assortative

We have selected all the subclusters having at least 30 amino acid nodes [12,13]. At *I*_*min*_ = 0 %, the all range (ARN), long-range (LRN) and short-range (SRN) interaction networks have positive (*r*)-values. The respective averages are 0.30, 0.17 and 0.21 (Table 1). Thus, it is very much clear that networks formed at different length scales of primary structure have assortative mixings of amino acid nodes. ARNs are composed of LRNs and SRNs. Thus, mixing behaviour of amino acids in overall protein contact network is contributed by both the LRN and SRN.

#### Mixing behaviour of amino acids depends on the type of residues

At *I*_*min*_ = 0%, the 91% of LRN-BNs clusters show assortative mixing; where average size of each cluster is 102 amino acid residues and the average value of $\left(r_{\mathrm{LRN}}^{b}\right)$ is 0.13 (Table 1). Both LRN-BNs and LRN-ANs show high number of assortative subclusters even at higher *I*_*min*_ cutoffs. On the other hand, most of the LRN-INs show disassortative mixing behavior with only 39% of the INs showing assortative mixing ($(r_{\mathrm{LRN}}^{i})∼$ -0.04), average size of the clusters at *I*_*min*_= 0% cutoff is 44 residues. The Mann-Whitney *U*-test shows that the average assortativity value of LRN-INs is significantly less than that of LRN-BNs(p-value = 3.553e-15). The LRN-CNs do not have any cluster having 30 or more nodes. The higher assortativity (or cluster size or clustering coefficients) of the BN subclusters than their respective IN subclusters, is independent of the number of hydrophobic or hydrophilic residues present in a protein. In our data set, 49 proteins have more number of hydrophilic residues than hydrophobics; even then the hydrophobic networks have larger average cluster size (BN∼ 146.79 and IN ∼ 118.18; p-value = 0.005) and a significantly higher assortativity (*r*^*b*^∼ 0.28 and *r*^*i*^∼ 0.18; p-value = 2.686e-06). The larger cluster sizes or assortativity values of the BNs thus indicate that these topological parameters depend on the physic-chemical behavior of constituent amino acids networks within the network.

Unlike LRNs, most of the SRN-BNs (almost 57%) show disassortative mixing of nodes. Average size of SRN-AN and SRN-BN clusters at 0% cutoff is about 431 and 39 amino acid residues, respectively.

ARNs are composed of LRN and SRNs, each of them show assortative mixing behavior. Again, each of these three networks has been classified into three different subnetworks based on their physico-chemical properties. In our earlier work (studied at *I*_*min*_=0% only) we have shown that the ARN-BNs exhibit assortative mixing properties. In addition, here, we observe that (i) the higher percentage of hydrophobic residues’ mixing behavior is of assortative type in LRN, and (ii) in SRN, the assortativity is an emergent property which is not apparently observed in its subclusters. Thus, the present result also confirms that the mixing behavior which also imply the connectivity pattern of the amino acid residues, depend on the physic-chemical nature of amino acids. Further, the propensity of an amino acid to be connected with other amino acids also depends on the position of the interacting amino acids in the primary structure. The mixing behavior of amino acids in overall protein and in long-range networks is more influenced by the hydrophobic residues.

#### Importance of assortative networks in communicating information

The allostery signals in proteins transmit from the perturbed effector site to the substrate site through pathways and the experimental data suggests that the allosteric pathways are highly populated with hydrophobic residues in some of the allosteric proteins. For example, Ranganathan and coworkers have predicted and confirmed experimentally a set of energetically coupled residues (which form the allosteric pathways for PDZ domain family); most of the residues in these pathways are hydrophobic [34]. A hydrophobic groove is also reported in the allosteric pathways of CREB binding protein CBP [35].

It is known that the information can be easily transferred through an assortative network as compared to a disassortative network [29]. We observe that most of the hydrophobic residues’ subnetworks in PCNs (LRNs and ARNs) are assortative in nature. Thus, one can expect that for any perturbation at the residue level, the necessary communication to the distantly located site would pass easily through the chain of hydrophobic residues. We should mention that our contact network is based only on London van der Waals interaction, we have not considered other type of non-covalent interaction (like electrostatic interaction between charged residues, or hydrogen bonds). However, the result of our simple model indicates that the necessary signal of perturbation can be easily communicated through hydrophobic networks due to their assortative mixing patterns.

Further, protein folding is a cooperative phenomenon, and hence, communication amongst amino acids is essential, so that appropriate non-covalent interactions can take place to form the stable native state structure [36]. Selvaraj and Gromiha [17] have shown that the hydrophobic clusters and network of long-range contacts pave the way for the folding and stabilization of alpha/beta barrel proteins. In another work [37], they have computed the hydrophobicity associated with each residue in the folded state and compared the Phi values of each mutant residues for a set of proteins and their results indicate the importance of hydrophobic interactions in the transition state. Considering the long-range contacts within proteins, Gromiha *et al* have introduced a parameter long-range Order (LRO) which correlates significantly with protein folding rate [38]. It is also reported that the assortativities in ARNs and LRNs positively correlate to the rate of folding [21]. While the previous studies indicate about the presence of long-range hydrophobic network in the folding transition state of proteins and positive correlation between long-range network parameter (LRO, assortative mixing) and folding rate of a protein, none has addressed the communication ability of information through the network. During in vivo protein folding, it is also very necessary to communicate the information as quickly as possible. Here, we show that the hydrophobic subclusters have the highest assortative mixing behavior in LRN and ARNs; and thus may indirectly indicate that the hydrophobic residues play an important role in communicating necessary information across the network in the folding process of a protein and help in determining the topology of tertiary structure of a protein. We should mention that this indication is just a hypothesis based on an indirect observation; the real picture can be captured by studying a competitive folding.

We next study the local cohesiveness of protein structures in terms of clustering coefficients and cliques of k=3.

### Clustering coefficients of subnetworks and their effects in protein folding and stability

Clustering coefficient is a measure of the cliquishness of a network. The average values of clustering coefficients (〈*C*〉) for long, short and all-range protein contact networks at *I*_*min*_= 0% are listed in Table 1. The average clustering coefficients of hydrophobic subclusters (〈*C*^*b*^〉) is the highest (even higher than that of all residues network) in both ARNs and LRNs. In deed, in LRNs, the average value of hydrophobic subclusters ($\langle C_{\mathrm{LRN}}^{b}\rangle$) is almost 1.5 times and double to those of all amino acids subcluster ($\langle C_{\mathrm{LRN}}^{a}\rangle$) and hydrophilic subclusters ($\langle C_{\mathrm{LRN}}^{i}\rangle$), respectively ( p-value < 2.2e-16). No charged subcluster with required number of nodes has been observed.

We know that the higher value of clustering coefficient of a node *i* indicates the higher number of connections among its neighbors (directly connecting nodes). The higher values of 〈*C*〉 in LRN-BNs and ARN-BNs than those of LRN-ANs and ARN-ANs, respectively, suggest that hydrophobic residues with higher clustering values interact in a more connected fashion, stitching different secondary, super-secondary structures and stabilizing the protein structure at the global level.

While the folding of a protein and attainment of the native 3D structure is stabilized by the long-range interactions [17], the clustering coefficients of LRNs show a negative correlation with the rate of folding of the proteins [21]. Understandably, more time is needed for more number of mutual contacts of long-range residues (higher clustering coefficients) for attaining the native state and hence, slower is the rate of folding. Thus it is expected that the higher values of clustering coefficients of a sub network indicate a larger effect on the part of its nodes (residues) in slowing down the rate of folding and helping in local structural organization. Thus, the higher average clustering coefficients of hydrophobic residues suggest higher contribution of hydrophobic residues in the folding rate of a protein.

### Occurrence of cliques

The clustering coefficient, 〈*C*〉 enumerates number of loops of length three. These loops (cliques) of length three can be generated by all possible combination of hydrophobic (B), hydrophilic (I) and charged (C) residues at the vertices of a triangle. Cliques are the subgraphs where every pair of nodes have an edge. In the previous section, we have only focused on BBB, III and CCC loops while studying the BNs, INs and CNs separately. Here, we have considered and calculated all the cliques that can be formed from the possible combination of hydrophobic, hydrophilic and charged residues (BBB, BBI, BBC, BII, BCC, BCI, CCC, III, CII, CCI).

The number of occurrences of all possible combination of cliques has been compared. For each protein, we have normalized the number of occurrences of the BBB or BCI (or others) cliques against the number of hydrophobic/hydrophilic/charged residues present in the protein. For example, a protein 1A2O has 173 hydrophobic residues and 939 BBB cliques, then we normalize the number of BBB cliques by diving it (939) by the number of all possible cliques that can be formed from the combination of 173 hydrophobic residues, and the new normalized value is 0.0011. The clique type with highest normalized clique occurrence value is identified for all the proteins. The relative frequency distribution (in %) of the clique types for ARN, LRN and SRN is shown in Additional file 4A. As quite expected, nearly 98% of proteins show highest number of BBB cliques in LRN-ANs and ARN-ANs,in while SRN-ANs, maximum number of proteins either have highest number of CCC loops (40.20%) or have highest occurrence of of BBB loops (33.73%). With increase in *I*_*min*_ cutoff, the subnetworks show a very interesting trait irrespective of length scale or type. The percentage of charged residues cliques increase with increase with *I*_*min*_cutoff. The frequency of occurrence of CCC loops is consistently followed by the CCI loops in all subnetwork types (Additional file 4B). These observations indicate that the charged residues loops (in addition to the hydrophobic loops) within a protein play important role in protein’s structural organization.

**Figure 3:**
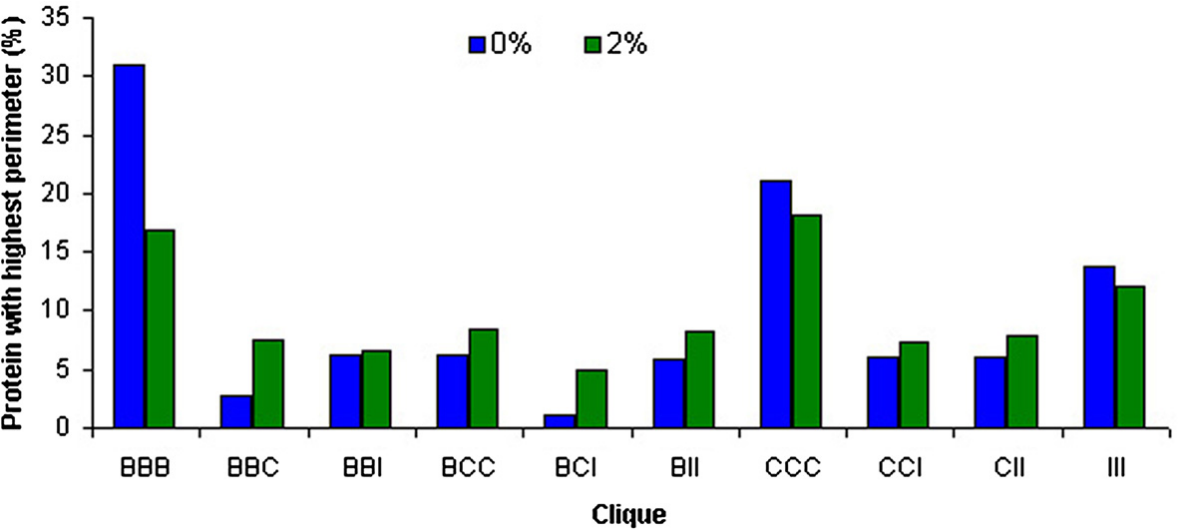
**The percentage of proteins for each clique type that covers maximum perimeter at 0% and 2%** ***I***_***min***_**cutoffs.** The average values of the perimeters for each clique type ARN-ANs and LRN-ANs are calculated. The number of times a clique type appears to have the maximum average perimeter value is expressed in terms of relative percentage of proteins for each clique type. The sum of all relative values of different clique types at each *I*_*min*_cutoff is 100.

To quantify how much distantly placed amino acid residues of primary structure form the vertices of a clique, we have used the perimeter of the clique (Additional file 5). The length of each side (edge between amino acid nodes) of a clique is basically the corresponding side (edge) forming amino acid’s distance in the primary structure. Higher perimeter of a clique implies more distantly placed residues in primary structure have come closer and making contacts in 3D space, thus playing an important role in fixing the tertiary structures. For each protein, we have calculated the average values of the perimeters for each type of combination of the cliques in ARN-ANs and LRN-ANs. Next, we identified the cliques with maximum values of average perimeters, and counted the number of times each clique type has the maximum average perimeter values. Next, we expressed the count of each clique type in terms of relative percentage i.e. if the count of BBB cliques having highest average perimeter value is 153 (out of total 495 proteins), its relative percentage is 30.90%. The relative percentage of each clique type is calculated and shown in Figure 3. As expected, BBB residues cliques cover maximum perimeters in 31% of proteins. Interestingly, the perimeters of all charged residues’ cliques (CCC) are maximum in approximately 21% of the proteins. In 11% proteins, hydrophilic loops (III) appear to cover maximum perimeter. Rest of the cliques which have non-similar residues vertices (BCC, BCI, BBC etc), do not show significant preference of any one over the others.

The occurrences and perimeters covered by cliques makes two clear observations. The first one confirms the well known information about the role of hydrophobic residues in tertiary structure formation. But the novel information which is coming out using the network analysis is that charged residue cliques have a higher strength of interaction among themselves, and that even though fewer in number, the charged cliques definitely bring the distantly placed amino acid residues along a polypeptide chain closer in the 3D space; thus helping in protein’s structural organization.

Comparing the transition of largest cluster size of real proteins with random model, Vishveshwara *et al* have concluded that the bond percolation resembles with random model (the probability of connection between two amino acids depends only on a specific *I*_*min*_); however clique percolation cannot be achieved by random like behaviour [39,40]. Thus, the presence of cliques and their properties are not random; rather they are related to the protein’s structural need. However, they have not addressed whether there is any preference of clique of specific amino acid residues. So far our knowledge, no previous study has addressed to compare the perimeter of the cliques. The results based on the perimeters of cliques clearly indicate the importance of charged residues (in addition to hydrophobic) in forming triad of distantly placed segments of primary structures in 3D space.

## Conclusions

The information regarding the tertiary structure of a protein is imprinted in the linear arrangement of its constituent amino acids and the said structure has evolved through interactions of amino acids in 3D space. Here, we have analyzed a large number of protein structures with a simple but powerful framework of protein contact network. Our results show that the method can extract several known properties of protein structure as well as can unravel several new features. The existence of comparatively larger size of LRN-LCC at higher interaction strength cut-off in thermophiles than mesophiles indicate that the higher interaction strengths among the amino acid nodes of these thermophilic long-range clusters provide extra stabilizing force to their tertiary structure. All the different length scale protein contact subnetworks have assortative mixing behavior of the amino acids. While the assortativity of long-range is mainly governed by their hydrophobic subclusters, the short-range assortativity is an emergent property not reflected in further subnetworks. The assortativity of hydrophobic subclusters in long-range and all-range network implies the quicker communication ability of hydrophobic subclusters over the others. We further observe the higher occurrences of hydrophobic cliques with higher perimeters in ARNs and LRNs. In SRNs, charged residues cliques have highest occurrences. In ARNs and LRNs, the percentage of charged residues cliques goes up with increase in interaction strength cutoff. This reflects that charged residues clusters (not just a pair of interaction), in addition to hydrophobic ones, play significant role in stabilizing the tertiary structure of proteins. Further, the assortativity and higher clustering coefficients of hydrophobic long-range and all range subclusters postulate a hypothesis that the hydrophobic residues play the most important role in protein folding; even it controls the folding rate. Finally, we should clearly mention that our network construction explicitly considers only the London van der Waals force among the residues. This does not include electrostatic interaction between charged residues or H-bonding, etc. To get further insights, one should explicitly consider all the non-covalent interactions among amino acids. However, it is interesting to note that the present simple framework of protein contact subnetworks is able to capture several important properties of proteins’ structures.

## Abbreviations

PCN, Protein contact network; SRN, Short-range interaction network; ARN, All-range interaction network; BN, Hydrophobic network; IN, Hydrophilic network; CN, Charged network; LCC, Largest connected component; Imin, Interaction strength cutoff; Icritical, Critical interaction strength; CI, Cooperativity index; r, Pearson correlation coefficient; C, Clustering coefficient.

## Competing interests

The authors declare that they have no competing interests.

## Author’s contributions

SK designed the experiment, DS performed the whole study and both of them prepared the manuscript. Both authors read and approved the final manuscript.

## Supplementary Material

Additional file 1 PDB codes of the 495 proteins used in the study. Click here for file

Additional file 2 Transition profiles of largest cluster in different subnetworks are compared for 495 proteins. The size of largest connected component is plotted as a function of *I*_*min*_ in different subnetworks for 495 proteins. The cluster sizes are normalized by the number of amino acid in the protein. The different subnetworks are A) Long-range all residue network (LRN-AN). B) Short-range all residue network (SRN-AN). C) All-range all residue network (ARN-AN). D) All-range hydrophobic residue network (ARN-BN). E) All-range hydrophilic residue network (ARN-IN). F) All-range charged residue network (ARN-CN). G) Long-range hydrophobic residue network (LRN-BN). H) Short-range hydrophobic residue network (SRN-BN). Click here for file

Additional file 3 Different nature of cluster in ARN-AN, LRN-AN and SRN-AN. The nature of cluster in SRN-AN is chain like while the cluster is much more well connected and non-chain like in LRN-AN and ARN-AN. Click here for file

Additional file 4 Relative highest frequency distribution in ARN, LRN and SRN. A. The number of occurrences of possible combination of cliques are normalized against the number of hydrophobic/hydrophilic/charged residues present in the protein. The frequency distribution (in %) of the clique types with highest normalized clique occurrence value is plotted for ARN, LRN and SRN at 0% *I*_*min*_ cutoff. The sum of all relative values of different clique types for each sub-network type is 100. B. The percentage of charged residues cliques increase with the increase in *I*_*min*_cutoff. This trend is followed at all length-scales. The sum of all relative values of different clique types at each *I*_*min*_cutoff is 100. Some sub-network types are not shown in the figure since they have a very less or no relative occurrence value. Click here for file

Additional file 5 Illustrative figure explaining perimeters of cliques. Higher perimeter of cliques means amino acids placed more distantly in primary structure come close in 3D space. So these residues must be of high importance in protein structure formation. Click here for file
