# Supplementary material for: Role of long- and short-range hydrophobic, hydrophilic and charged residues contact network in protein’s structural organization
Source: BMC Bioinformatics. 2012 Jun 21;13:142. doi: 10.1186/1471-2105-13-142 (PMC3464617; doi:10.1186/1471-2105-13-142)
Supplement: Additional file 1 — PDB codes of the 495 proteins used in the study. [file 1471-2105-13-142-S1.pdf]

12AS, 1A0C, 1A12, 1A2O, 1A99, 1AC5, 1AD3, 1AOR, 1BGV, 1C1D, 1CCW, 1CG2, 1CII, 1CIY, 1CKM, 1CQX, 1CRU, 1CVR, 1CWV, 1D0C, 1DDZ, 1DL5, 1DQ3, 1DS1, 1DUV, 1ECF, 1EDZ, 1EEX, 1EHI, 1EJD, 1EU8, 1EVL, 1EZW, 1F1U, 1FGJ, 1FOB, 1G5A, 1G9G, 1GA6, 1GHQ, 1GKM, 1GNL, 1GP6, 1GSA, 1GVE, 1GWU, 1H12, 1H16, 1H6D, 1HCI, 1HM9, 1HT6, 1HX6, 1HYN, 1HYO, 1I5P, 1IDK, 1IHG, 1IOM, 1IOW, 1ITB, 1IZO, 1J5Q, 1JA1, 1JET, 1JF9, 1JIX, 1JKM, 1JR3, 1JZ8, 1K5C, 1K7W, 1KHV, 1KKH, 1KP8, 1KQ3, 1L0W, 1L5J, 1L5O, 1LF6, 1LFW, 1LLD, 1LNS, 1LZL, 1M1J, 1M1N, 1MDL, 1MV8, 1MXR, 1N62, 1N7O, 1ND6, 1NOF, 1NTH, 1NVM, 1NW3, 1NY5, 1OAO, 1OCK, 1OGO, 1OGQ, 1OHE, 1OLM, 1OMO, 1ORR, 1OXX, 1OZ2, 1PBY, 1PE9, 1PGL, 1PGS, 1PJX, 1PPJ, 1PPR, 1Q0Q, 1Q8F, 1QAZ, 1QD1, 1QHD, 1QI9, 1QOY, 1QSA, 1R17, 1R1H, 1R31, 1R6D, 1R9D, 1R9L, 1RA0, 1REQ, 1RJD, 1RK6, 1RKD, 1RM6, 1RU4, 1RWH, 1RYI, 1S16, 1S9R, 1SB8, 1SBP, 1STZ, 1SYY, 1T1U, 1T2D, 1T5J, 1T5O, 1T77, 1TBF, 1TG7, 1TV8, 1TWI, 1TXG, 1U1I, 1U3D, 1U4Q, 1U5U, 1U60, 1UF5, 1UHV, 1US0, 1UUQ, 1V5V, 1VCL, 1VE9, 1VLB, 1VLJ, 1VPK, 1VR6, 1VSY, 1W07, 1W0P, 1W23, 1W3B, 1W55, 1W61, 1W6S, 1W79, 1WDP, 1WER, 1WKR, 1WM1, 1WMW, 1WPG, 1WVF, 1WYU, 1X38, 1X3L, 1X54, 1XCR, 1XFK, 1XME, 1XO0, 1XOC, 1XPM, 1XRS, 1XSZ, 1Y3T, 1Y4W, 1Y8A, 1YDY, 1YEW, 1YFQ, 1YGA, 1YKD, 1YQD, 1YQZ, 1Z2N, 1Z3I, 1Z5H, 1ZDY, 1ZGS, 1ZK7, 1ZL0, 1ZQ1, 1ZU0, 1ZXX, 1ZZ1, 1ZZG, 2A1H, 2AC1, 2AEU, 2AHF, 2ALA, 2AQJ, 2AZ4, 2B0T, 2B3F, 2B5E, 2B5U, 2BB6, 2BJF, 2BJQ, 2BVF, 2C1L, 2C1V, 2C31, 2C6Q, 2C7P, 2CAS, 2CN3, 2D0O, 2D3A, 2DDX, 2DE6, 2DF7, 2DFK, 2DG1, 2DKJ, 2DRW, 2DVT, 2DW0, 2DY1, 2E4T, 2E5F, 2ELC, 2EX0, 2EZ2, 2F7F, 2FBA, 2FGQ, 2FHF, 2FJI, 2FP4, 2FP8, 2FQ6, 2FQX, 2GFI, 2GUY, 2GWD, 2H1V, 2H1Y, 2H2Z, 2H6F, 2HBV, 2HC9, 2HCY, 2HDW, 2HEU, 2HNH, 2HOX, 2HWK, 2HZG, 2I06, 2I0Q, 2I2X, 2I9U, 2INP, 2IUT, 2IVF, 2IVN, 2IXS, 2J4O, 2J6L, 2JBV, 2JD4, 2JEP, 2JGP, 2JLQ, 2NQL, 2NT0, 2NVO, 2NZX, 2O4C, 2O5V, 2OBD, 2OKT, 2OKX, 2ONS, 2OSX, 2OXN, 2OZL, 2P0W, 2PBI, 2PFZ, 2PIA, 2PO3, 2POK, 2POR, 2Q01, 2QAP, 2QHF, 2QMC, 2QQI, 2QRL, 2QY1, 2QZS, 2R2N, 2R60, 2R6J, 2RA1, 2RFT, 2UVJ, 2UW1, 2UYT, 2V3A, 2V8Q, 2VGL, 2VPZ, 2VSG, 2VZS, 2W40, 2W8S, 2W9M, 2WIY, 2WJ1, 2WNH, 2WNW, 2WQ7, 2WVG, 2WW5, 2WYA, 2WZP, 2X3H, 2X49, 2XFN, 2XM5, 2YHX, 2Z5G, 2ZD1, 2ZGY, 2ZHJ, 2ZJ8, 2ZSJ, 2ZUM, 2ZUX, 2ZY4, 2ZYJ, 2ZYR, 2ZZV, 3A04, 3A09, 3A16, 3A4V, 3A72, 3ABI, 3AEK, 3AHC, 3AHN, 3B34, 3B9O, 3BEC, 3BG1, 3BH7, 3BJE, 3BMA, 3BNJ, 3BOF, 3BPW, 3BQW, 3BZ5, 3BZC, 3C46, 3C7A, 3C7F, 3C9U, 3CBW, 3CLW, 3CQD, 3CRV, 3CTZ, 3CWN, 3CX5, 3D59, 3D5K, 3DG6, 3DJL, 3DRF, 3DSD, 3DW8, 3DWN, 3E0M, 3E2D, 3E4W, 3E7J, 3EDF, 3EDY, 3ETC, 3EUH, 3F47, 3FR7, 3FVZ, 3FWN, 3GD6, 3GE3, 3GKE, 3GKR, 3GR4, 3GWJ, 3GZK, 3H12, 3H5L, 3H63, 3H6G, 3HFW, 3HG7, 3HHD, 3HHP, 3HHW, 3HJH, 3HJL, 3HR8, 3I45, 3I5X, 3I83, 3IAG, 3IAV, 3IJ6, 3IJL, 3ILW, 3IM9, 3IP4, 3IRP, 3IX1, 3JS8, 3JSL, 3JTM, 3JYH, 3K26, 3K4K, 3K7M, 3K9V, 3KB9, 3KCP, 3KEF, 3KIC, 3KKI, 3KLK, 3L7I, 3L8Q, 3LE2, 3LGD, 3LSS, 3LWT, 3LXY, 3LY0, 3M73, 3M7V, 3MKH, 3MW9, 3N6O, 3NA0, 3NDI, 3NEH, 3NRZ, 3NVA, 3NYB, 3NYT, 3O2G, 3O8M, 3O9Z, 3OC9, 3OKP, 3OR1, 3OY9
