# Supplementary figures and images for: Role of long- and short-range hydrophobic, hydrophilic and charged residues contact network in protein’s structural organization
Source: BMC Bioinformatics. 2012 Jun 21;13:142. doi: 10.1186/1471-2105-13-142 (PMC3464617; doi:10.1186/1471-2105-13-142)

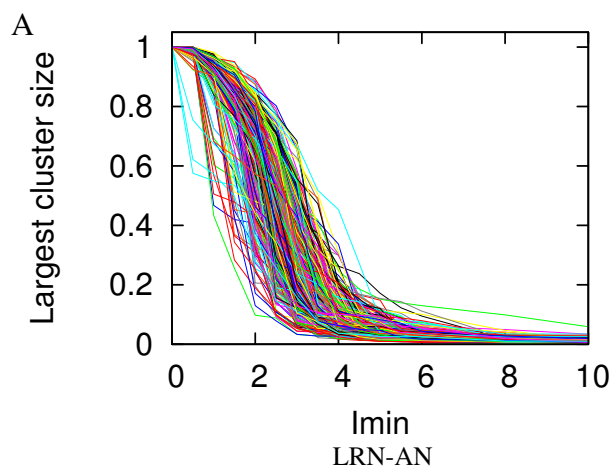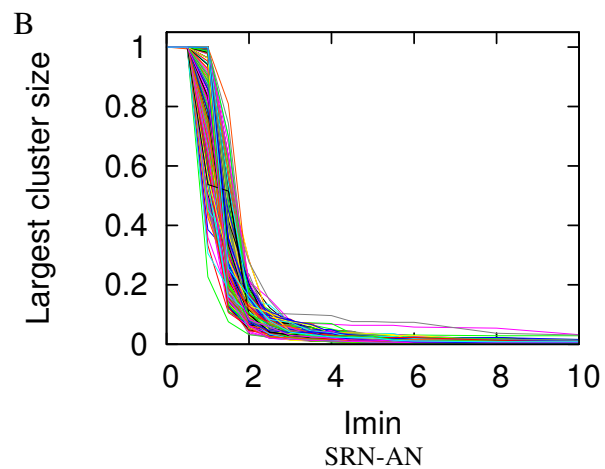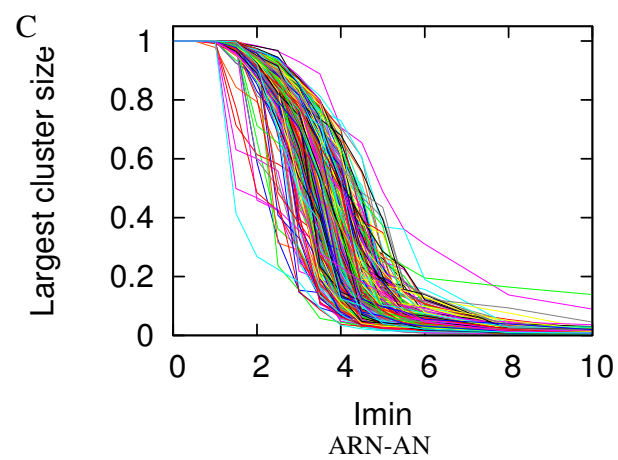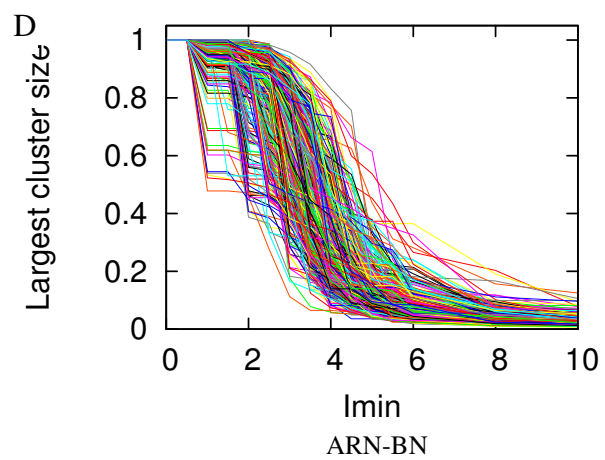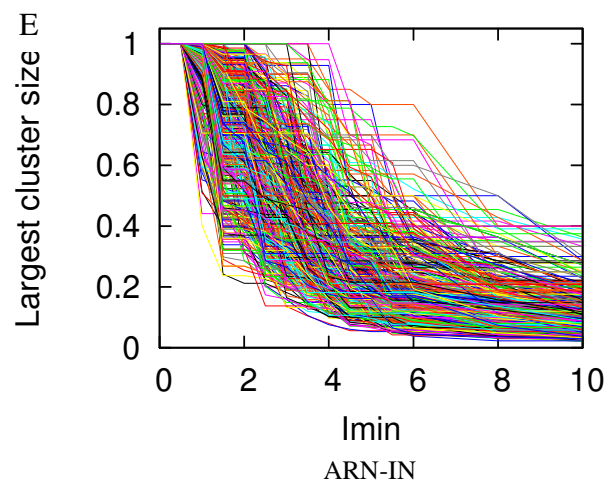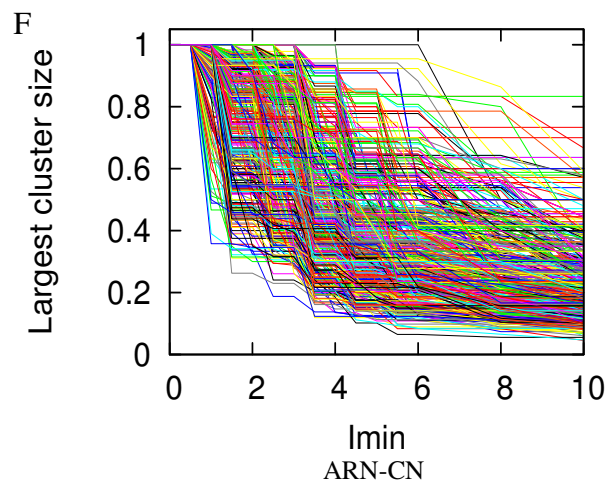

G

Largest cluster size

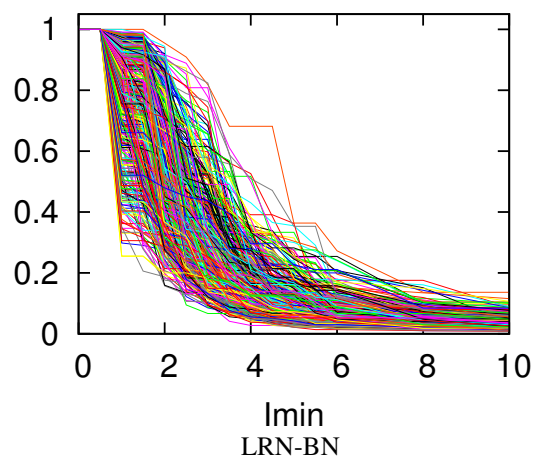

H

Largest cluster size

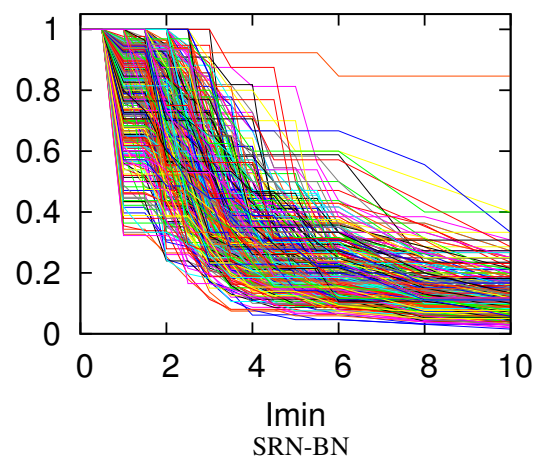

Supplement: Additional file 2 — Transition profiles of largest cluster in different subnetworks are compared for 495 proteins. The size of largest connected component is plotted as a function of I min in different subnetworks for 495 proteins. The cluster sizes are normalized by the number of amino acid in the protein. The different subnetworks are A) Long-range all residue network (LRN-AN). B) Short-range all residue network (SRN-AN). C) All-range all residue network (ARN-AN). D) All-range hydrophobic residue network (ARN-BN). E) All-range hydrophilic residue network (ARN-IN). F) All-range charged residue network (ARN-CN). G) Long-range hydrophobic residue network (LRN-BN). H) Short-range hydrophobic residue network (SRN-BN). [file 1471-2105-13-142-S2.pdf]

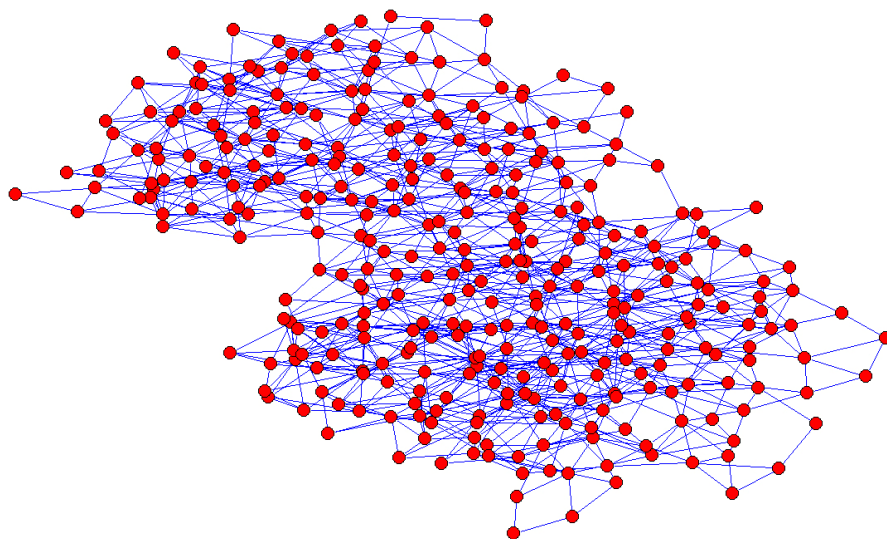

ARN-AN cluster

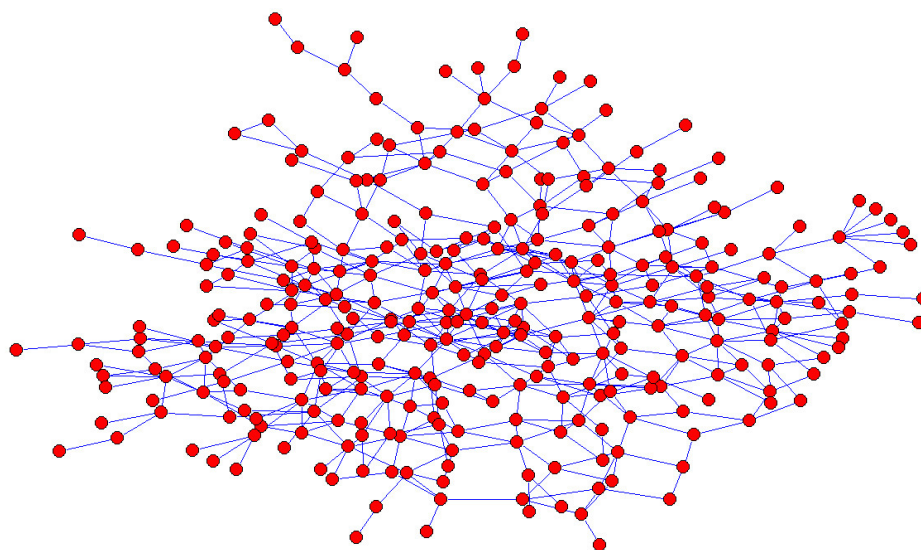

LRN-AN cluster

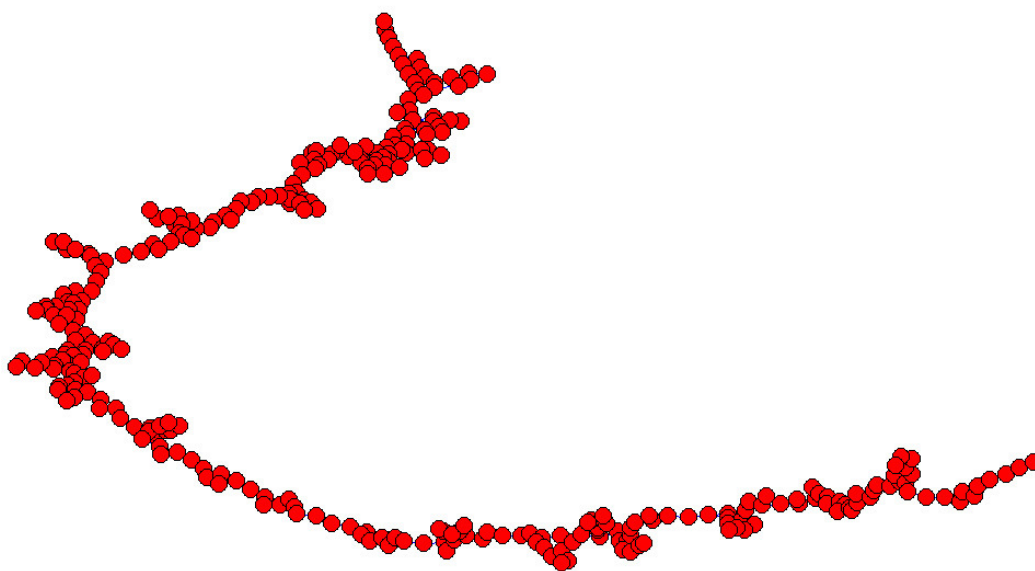

SRN-AN cluster

Supplement: Additional file 3 — Different nature of cluster in ARN-AN, LRN-AN and SRN-AN. The nature of cluster in SRN-AN is chain like while the cluster is much more well connected and non-chain like in LRN-AN and ARN-AN. [file 1471-2105-13-142-S3.pdf]

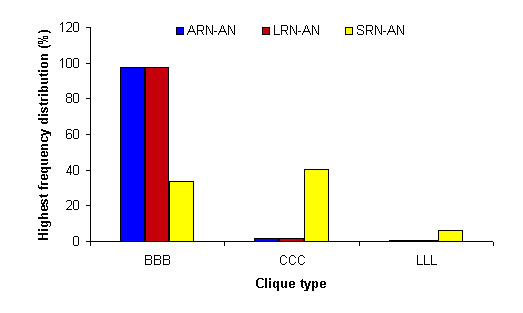

Supplement: Additional file 4 — Relative highest frequency distribution in ARN, LRN and SRN. A. The number of occurrences of possible combination of cliques are normalized against the number of hydrophobic/hydrophilic/charged residues present in the protein. The frequency distribution (in %) of the clique types with highest normalized clique occurrence value is plotted for ARN, LRN and SRN at 0% I min cutoff. The sum of all relative values of different clique types for each sub-network type is 100. B. The percentage of charged residues cliques increase with the increase in I min cutoff. This trend is followed at all length-scales. The sum of all relative values of different clique types at each I min cutoff is 100. Some sub-network types are not shown in the figure since they have a very less or no relative occurrence value. [file 1471-2105-13-142-S4.jpeg]

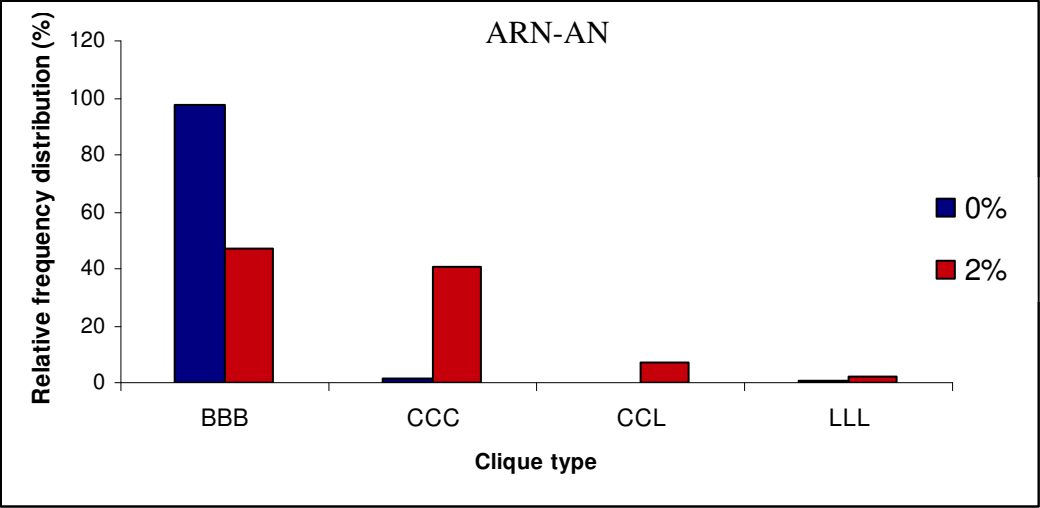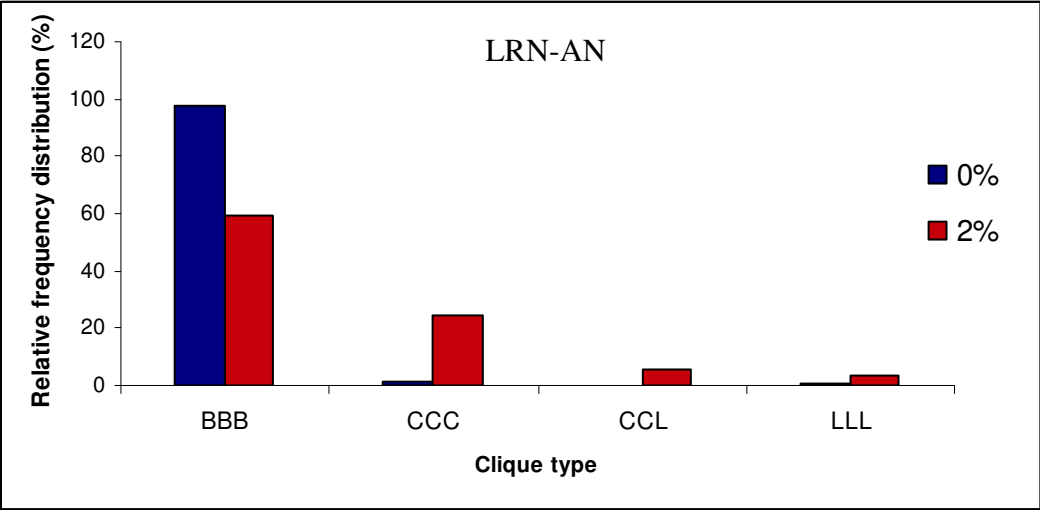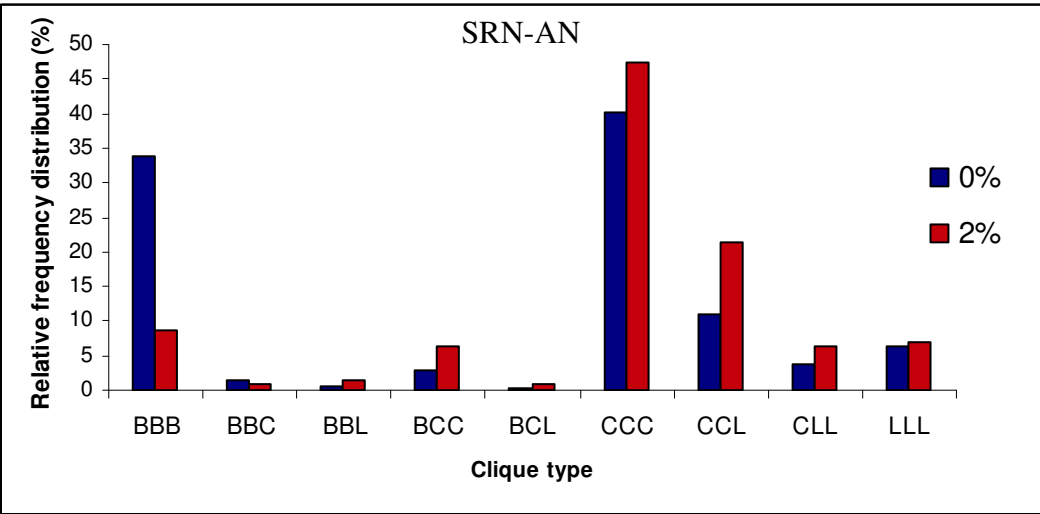

Supplement: Additional file 5 — Illustrative figure explaining perimeters of cliques. Higher perimeter of cliques means amino acids placed more distantly in primary structure come close in 3D space. So these residues must be of high importance in protein structure formation. [file 1471-2105-13-142-S5.pdf]
